# Registering Study Analysis Plans (SAPs) Before Dissecting Your Data—Updating and Standardizing Outcome Modeling

**DOI:** 10.3389/fonc.2020.00978

**Published:** 2020-06-24

**Authors:** Maria Thor, Jung Hun Oh, Aditya P. Apte, Joseph O. Deasy

**Affiliations:** Department of Medical Physics, Memorial Sloan Kettering Cancer Center, New York, NY, United States

**Keywords:** cancer, clinical trial, observational study, outcome modeling, preregistration, public repository, radiotherapy, study plan

## Abstract

Public preregistration of study analysis plans (SAPs) is widely recognized for clinical trials, but adopted to a much lesser extent in observational studies. Registration of SAPs prior to analysis is encouraged to not only increase transparency and exactness but also to avoid positive finding bias and better standardize outcome modeling. Efforts to generally standardize outcome modeling, which can be based on clinical trial and/or observational data, have recently spurred. We suggest a three-step SAP concept in which investigators are encouraged to (1) Design the SAP and circulate it among the co-investigators, (2) Log the SAP with a public repository, which recognizes the SAP with a digital object identifier (DOI), and (3) Cite (using the DOI), briefly summarize and motivate any deviations from the SAP in the associated manuscript. More specifically, the SAP should include the *scope* (brief data and study description, co-investigators, hypotheses, primary outcome measure, study title), in addition to step-by-step details of the *analysis* (handling of missing data, resampling, defined significance level, statistical function, validation, and variables and parameterization).

## Introduction

Starting from 1997, the Food and Drug Administration Modernization Act (FDAMA) mandated the National Institute of Health (NIH) to design a platform in which information about FDA regulated clinical trials would become publicly available^1^. As a result, NIH launched ClinicalTrials.gov shortly thereafter (1). Public pre-registration of clinical trials has since become a general publication requirement (2), and fast forwarded to two decades after FDAMA was introduced, ClinicalTrials.gov hosts 341 988 (as of June 11, 2020) registered studies conducted worldwide^2^.

**Figure 1:**
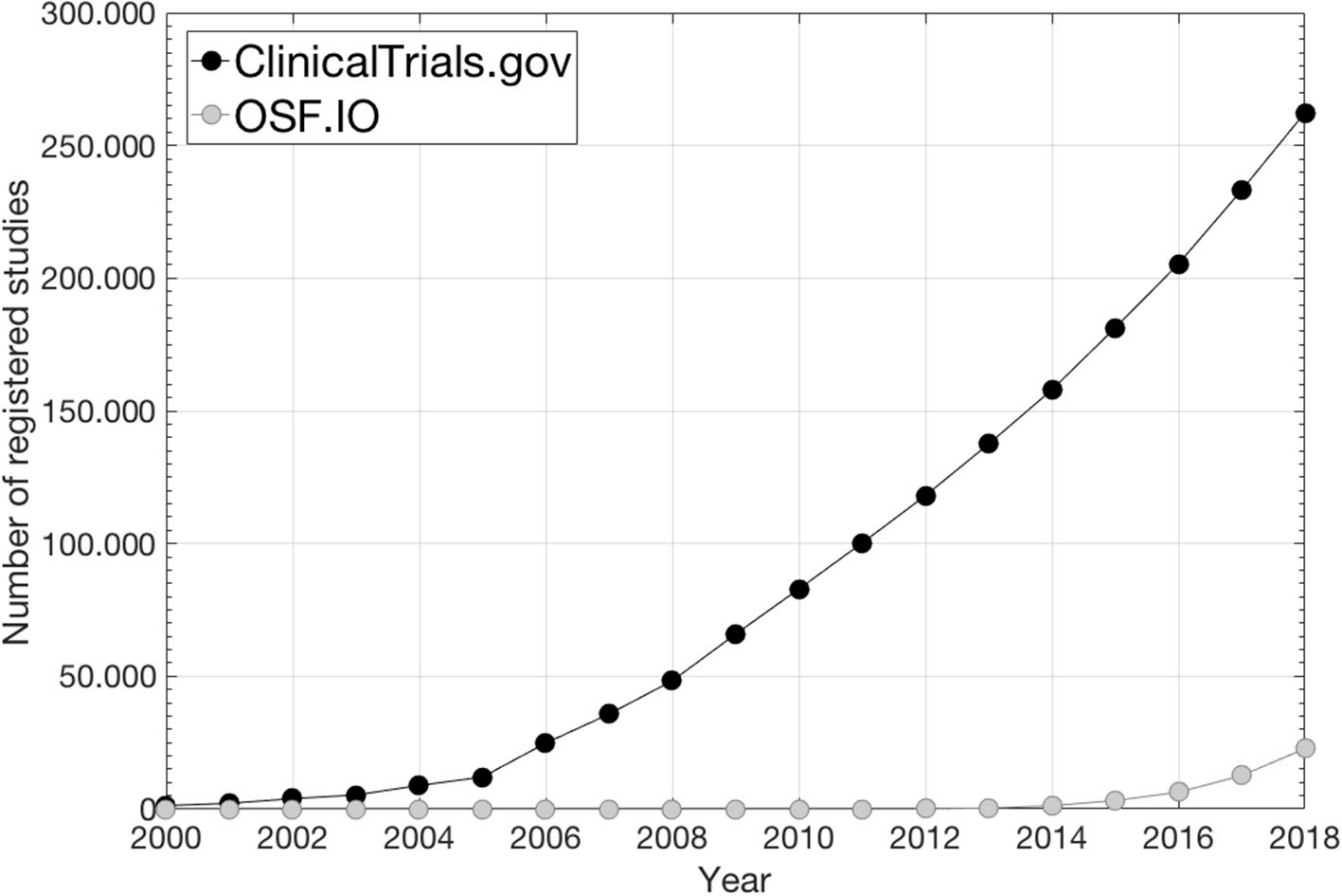
The number of preregistered SAPs during the last two decades under ClinicalTrials.gov^2^ and under OSF^3^ (data from ClinicalTrials.gov is taken from^2^; data from OSF is taken from https://cos.io/our-products/osf-registries/).

This site primarily focuses on interventional studies/clinical trials while study analysis plans (SAPs) and associated results from observational studies are scarce (1)^2^ as also illustrated in Figure 1 where the number of preregistrations from ClinicalTrials.gov and from the Open Science Foundation (OSF)^3^, which mainly holds SAPs from observational studies, is given over time. Consequently, for observational exploratory research it is often unclear as to the number of analyses undertaken, which further feeds into what is referred to as “*p-hacking*,” i.e., a positive finding publication bias since the vast majority of published studies that report *p*-values disclose positive/significant findings (3, 4). Further, SAP pre-registration is likely to facilitate researchers to better distinguish between confirmatory research (hypothesis-testing in which *p*-values retain diagnostic value) and exploratory research (hypothesis-generating in which *p*-values loose diagnostic value) in order to avoid overconfidence in *post-hoc* explanations in a finding that has not been proven, which could limit reproducibility (5).

The Transparent Reporting of a multivariate prediction model for Individual Prognosis Or Diagnosis (TRIPOD) statement has encouraged to better standardize outcome modeling (6). Outcome modeling can be based on data generated from clinical trials or observational studies. Here we propose to pre-register SAPs under public repositories for any outcome modeling study to further promote standardization, transparency and exactness and to mitigate the false positive inflation of published results.

## Methods and Materials

Public pre-registration of SAPs could be thought of as committing to an analytical path but without advancing knowledge of the research outcome (4). To date, the two most commonly used public SAP repositories, which both provide SAP unique digital object identifiers (DOIs), are located under ClinicalTrials.gov (1) and under the OSF^3^. As previously pointed out, ClinicalTrials.gov has primarily been used to register clinical trials, while under OSF a larger extent of SAPs from observational studies can be found.

## The SAP Concept

**Figure 2:**
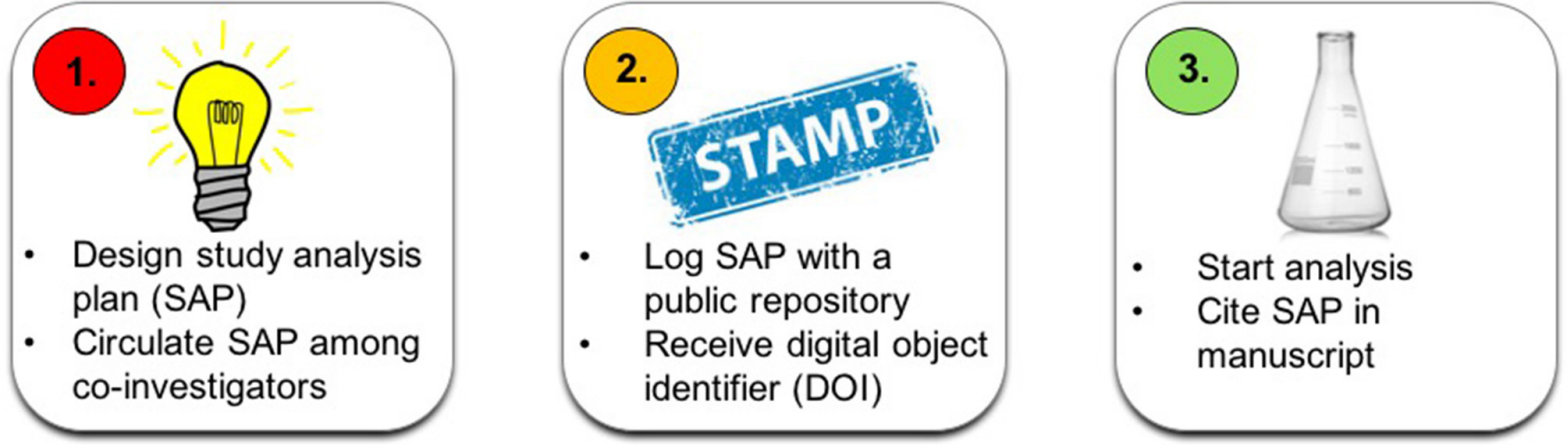
A flow chart of the suggested three-step SAP concept: (1) Design SAP and distribute to co-investigators (left); (2) Log SAP (middle), and (3) Cite (using the DOI), summarize and motivate any deviations from the SAP in the associated manuscript (right).

The suggested SAP concept consists of three steps: (1) Designing the SAP and circulating it among the co-investigators; (2) Logging the SAP with a public repository, which recognizes the SAP with a DOI, and (3) Citing (using the DOI), briefly summarizing and motivating any deviations from the SAP in the associated manuscript (*Note: any new major post-SAP analysis should only be considered hypothesis-generating/exploratory*). The three-step SAP concept is summarized in Figure 2.

The outcome modeling pipeline in the SAP should adhere to the modeling procedures defined in the TRIPOD landmark paper on how to model outcomes (6). This refers to description of data, outcomes and input variables and parameterization in addition to detailed step-by-step lay-out of the analysis. Below we list more specifically what the SAP should include (at a minimum) inspired by OSF's preregistration template, which is available as a GoogleDoc here: https://docs.google.com/document/d/1DaNmJEtBy04bq1l5OxS4JAscdZEkUGATURWwnBKLYxk/edit?pli=1, but more directed toward outcome modeling assuming an observational study design. An associated example SAP template for the purpose of outcome modeling is provided in the Supplementary Material.

### Scope (Description of Data and Study)

The study scope should include title, co-investigators, and a brief study description, and the underlying study hypothesis/hypotheses. The brief study description should be accompanied by a description of data/patient population (inclusion criteria, number of patients, primary tumor site, treatment era, etc.) and primary outcome measure with range and minimum follow-up time and censoring defined if applicable. The study type should also be clearly stated (e.g., validation, exploration, and/or prediction).

### Analysis (Description of the Analysis)

All variables considered for analysis should be described in detail along with their parameterization (binary, categorical and continuous; specify increments if applicable). Handling of missing data (if excluding data then describe how this will be accounted for) should be disclosed, and if applicable data transformation (or normalization) as well as definition of variable interaction terms should be given. The exact definition of the studied outcome, e.g., timing and scoring of radiation-induced toxicity and how pre-treatment status was taken into account, should be given. Although this SAP concept work focuses on outcome modeling in general, the expected minimum level of detail on reported variables is exemplified for RT dose, which is central for outcome modeling following RT: Specify if dose was parameterized as 2D dose-volume histograms (denote metrics, interval investigated and sampling), and/or summary measures such as the mean dose or the generalized equivalent uniform dose, and/or if being represented spatially (denote metrics and describe method) and if dose originated from the planned dose distribution, if being accumulated (plus type of dose accumulation), and/or if during treatment dose was used (applies possibly only for acute toxicity). Denote if and how fractionation effects were handled, and give the exact anatomical definition of the investigated organ(s) along with the associated segmentation approach. Please refer to the Results section for a practical example of the level of detail in describing dose.

The statistical functions/methods of analysis (e.g., regression (and type), time to event, competing risk, etc.) should be explained in detail along with a description of risk groups and defined errors/confidence intervals (if valid), any considered resampling (e.g., iterated cross-validation hold-out or bootstrapping; number of iterations, etc.), validation (external/internal), and if and how univariate and/or multivariate analysis will be performed. Any considered level of significance/model quality should be specified and the associated performance metric described. If investigating more than one variable authors should denote how multiple testing will be corrected for.

Lastly, the SAP should include the statistical software tools (and version) that are being considered.

## Literature Resources for Outcome Modeling in Radiotherapy

Aside from advocating the use of TRIPOD (6) as a guideline for outcome modeling in general, we below provide a short introduction to relevant literature for outcome modeling in radiotherapy (RT) with a particular emphasis on standardization.

To obtain reliable information about toxicities that influence patient's quality of life, normal tissue toxicities are likely best represented by patient-reported outcomes (PROs) (7). Using clinical decision-support tools (8, 9) and keeping the number of items/questions as few as possible (10) are necessary for actionability to patient-reported complaints. Dose-volume histogram (DVH) metrics of interest depend on a large variety of factors as pointed out within the 21 papers by the QUANTEC effort (11). Gathering published DVH metrics to better understand the reliability and generalizability of such metrics was first initiated by QUANTEC and is, as illustrated in their offspring efforts [pediatric RT (12) and hypo fractionated RT (13)] and work by other groups (14), a continuous process. These and related efforts (ideally multi-institutional) in which models have been validated (6, 15, 16) probably hold the most reliable DVH findings. Also, incorporation of additional sources of data is likely to shed much further light on the complex mechanisms of both tumor response and normal tissue toxicity following RT. Examples are shown in studies focusing on genome-wide assays (17) and immune status (18) as well as medical imaging within associated standardization efforts (19).

## Results

### A SAP Pre-registration Example

The authors recent experience in depositing an outcome modeling SAP with a public repository (15) will be used as an example of the SAP pipeline and content for outcome modeling.

After circulating the SAP among co-investigators, the SAP was logged with the OSF on July 23rd 2018 (15), and the analysis was, thereafter, initiated [the associated full-length manuscript was recently accepted for publication (16)].

As stated under the study *scope* (15), data were generated from a clinical trial (20) but the trial was not part of the outcome modeling itself. The *primary outcome measure* was overall survival defined from the start date of randomization and right-censoring was applied if alive at the last follow-up. For *Input data* related to disease, patient and treatment characteristics (the latter included 2D DVH parameterizations of the atria, lung, pericardium, and ventricles [please see (16) for exact anatomical definitions and parameterization of the remaining input data] structures: the minimum dose to the hottest 5–95% volume in steps of 5%, mean dose, minimum dose, max dose and the mean of the hottest 5–100% volume in steps of 5%, all metrics were corrected for fractionation effects assuming α/β = 3 Gy), significance was denoted at a 5% Bonferroni-corrected level. *Validation* was considered using a holdout subset on which performance would be assessed after settling the final model. The validation procedures were directly adopted from a previously published study (21). The main *statistical function* was Cox Proportional Hazard regression. Both univariate and multivariate analyses, with a clear advancement criterion (*p* < 0.05 of the log-likelihood statistics), were undertaken, and re-sampling was considered using Bootstrapping with 1,000 iterations. Lastly, arriving at the final model, two alternative approaches were explored—the ≥10% most frequently selected multivariate models or the ensemble thereof.

## Conclusion

We have suggested a SAP pre-registration pipeline to be used for outcome modeling studies, which typically use observational data. An example of an already submitted SAP and cited for outcome modeling is given along with an outcome modeling directed SAP template. The ambition of the authors is that pre-registration of SAPs, using the suggested layout and pipeline, is becoming standard, like it has for clinical trials, also in outcome modeling.

## Author Contributions

MT and JD contributed to the conception and design of the study. MT drafted the manuscript. All authors contributed to manuscript revision, read, and approved the submitted version.

## Conflict of Interest

The authors declare that the research was conducted in the absence of any commercial or financial relationships that could be construed as a potential conflict of interest.

## Footnotes

^1^Food and Drug Administration Modernization Act of 1997: https://www.govinfo.gov/content/pkg/PLAW-105publ115/pdf/PLAW-105publ115.pdf#page=16

^2^https://clinicaltrials.gov/ct2/resources/trends

^3^https://osf.io/
