# Supplementary material for: Registering Study Analysis Plans (SAPs) Before Dissecting Your Data—Updating and Standardizing Outcome Modeling
Source: Front Oncol. 2020 Jun 24;10:978. doi: 10.3389/fonc.2020.00978 (PMC7327097; doi:10.3389/fonc.2020.00978)
Supplement: Supplementary file 1 [file Data_Sheet_1.docx]

# Supplementary material

# Example SAP template for outcome modeling.

Project title

Study Analysis Plan (SAP id: XX)

First and last name of Principal investigator

Date of SAP deposited to public repository

# Scope

## Investigators

XX, YY, and ZZ

## Description of the data and study

### Include a brief description of the study and underlying hypotheses. Also, describe the patient population (inclusion criteria, number of patients, treatment era, tumor site, *etc.*) and define the primary outcome measure (if censoring, indicate if left/right and give censoring defenition). Lastly, denote the study type (clinical trial or observational study).

# Analysis

## Description of the analysis

Outline all variables to be considered and their parameterization (binary, categorical and continuous; specify increments if applicable). Disclose handling of missing data (if excluding data then describe how this will be accounted for), and any data transformation and consideration as well as definition of variable interaction terms. Describe the statistical functions considered, risk groups, errors/confidence intervals (if applicable), any considered resampling (*e.g.*, iterated cross-validation or bootstrapping; number of iterations, *etc.*), validation (external/internal), and if and how univariate and/or multivariate analysis will be performed. Specify any considered level of significance/model quality and the associated performance metric. If investigating more than one variable, denote how multiple testing will be corrected for. Lastly, give the name of the statistical software (and version) being used.

For further information regarding outcome modeling please refer to TRIPOD^1^.

# Bibliography

^1^ Moons KG, Altman DG, Reitsma JB, *et al*. Transparent Reporting of a multivariate prediction model for Individual Prognosis Or Diagnosis (TRIPOD): Explanation and Elaboration. *Ann Intern Med* 2015;162:1-73

^2^ …
